# Supplementary material for: Are carbon emissions trading and green financial instruments synergistic? -Comprehensive quantitative research based on content analysis
Source: PLoS One. 2024 Mar 7;19(3):e0298601. doi: 10.1371/journal.pone.0298601 (PMC10919601; doi:10.1371/journal.pone.0298601)
Supplement: S1 Data — (ZIP) [file pone.0298601.s001.zip › Data/地级市Z_F工作报告.zip!/╣π╢1⁄2/╓Θ║ú╩╨/└┤╫╘╣1⁄2╓┌║┼í╛┬φ┐╦╩2╛▌═°í┐_2010.docx]

各位代表：

　　我代表市人民政府向大会作政府工作报告，请予审议，并请政协委员和其他列席人员提出意见。

　　迎难而上、拼搏进取的2009年

　　2009年是改革开放以来我市经济发展最为困难、经济结构性矛盾暴露最为明显的一年，也是我市应对国际金融危机、经济社会发展取得显著成绩的一年。作为全省经济外向度最高的城市，我市受国际金融危机冲击尤为严重，企业订单大幅下降，出口明显下滑，企业生产经营异常艰难，经济工作面临空前的压力和挑战。我们在党中央国务院、省委省政府和市委的正确领导下，在市人大及其常委会和市政协的监督支持下，以实施《珠江三角洲地区改革发展规划纲要》（以下简称《规划纲要》）为主线，坚定不移推进珠江口西岸核心城市建设，全面落实保增长保民生保稳定和“三促进一保持”的要求，化危为机求突破，攻坚克难破瓶颈，着眼未来促转型，夯实基础定格局，在科学发展的道路上迈出了可喜步伐。

　　——经济发展取得新成绩。GDP突破千亿元大关达到1037.7亿元，在严峻的经济形势下，增长6.6％；预计人均GDP突破1万美元达到6.98万元，增长5.3％。固定资产投资额410.5亿元，增长10.3％。社会消费品零售总额413.8亿元，增长15％。城镇居民人均可支配收入22859元，增长9.1％，近十年来首次高于GDP增速；农渔民人均纯收入8575元，增长6.6％。接待游客2087.7万人次，增长9.1％。金融机构各项存款余额2105.2亿元，增长33.6％。来源于珠海的财税总收入305.4亿元；财政一般预算收入101.4亿元，增长9.9％。

　　——重大产业项目取得新突破。中船1100万吨级造船基地配套工程、中国海油深水工程基地、中航通用飞机产业基地和玉柴发动机等一批总投资1300多亿元的重大项目陆续动工，为抢占战略性新兴产业制高点、壮大产业规模奠定了基础。招商引资成绩骄人，引进内资注册资本金总额56.9亿元，增长88.8％。实际利用外商直接投资11.8亿美元，增长3.3％，比全国、全省平均增幅分别高出5.9和1.4 个百分点。中央和省属企业在珠海累计投资项目34个、总投资超过1300亿元。重大项目招商势头良好，协议总投资200亿元以上的项目2个、100亿元以上的6个、50亿元以上的7个。

　　——交通建设迈上新台阶。港珠澳大桥动工，进一步提升珠海的区位优势。广珠城际轨道和广珠铁路建设顺利推进，计划于今明两年先后竣工通车。高栏港高速、机场高速、井岸二桥以及珠海大道、柠溪路改造、板樟山隧道大修工程等动工建设。省道S272白蕉高速公路出口至湖心路口段建成通车。5万吨级集装箱码头第二个泊位竣工，10万吨级煤码头和两个15万吨级矿石码头动工建设，10万吨级集装箱码头和10万吨级进港主航道项目获得国家批准，珠海港成为珠三角地区水深条件最好、功能最齐全的港口。

　　——区域合作结出新硕果。国务院批准实施《横琴总体发展规划》，使横琴开发上升为国家战略，将横琴纳入珠海经济特区范围，想方设法落实横琴在通关制度、金融创新、产业和信息化、土地管理等方面的特殊政策。横琴新区专项政策和规划编制报批等工作加紧推进，多联供燃气能源站、长隆国际海洋度假区等一批项目动工建设。出台了推进珠港澳更紧密合作的实施意见，开展对港澳货物贸易人民币结算试点。珠澳合作开创新局面，与澳门特区政府达成珠澳同城化共识，建立官方正式联络机制，重点推进基建对接、通关便利、产业互补、服务一体等方面合作。千方百计应对咸潮，强化科学调度，加紧推进总库容4333万立方米的竹银水源工程，保障珠澳供水。召开首届珠中江区域合作联席会议，确定八大重点合作领域，20多项实质性合作项目加紧推进，实现公交互通、年票互认、媒体互动、环保联动，加快珠中江经济圈一体化进程。

　　一年来，我们主要抓了以下工作：

　　一、积极应对危机，经济回升向好发展

　　遏制下滑促增长成效明显。面对国际金融危机冲击带来的压力和挑战，市政府见事早、谋划准、出手快、出拳重，沉着应对，积极化危为机、危中求进。坚持把扩内需与稳外贸结合起来，把保增长与调结构结合起来，把惠民生与保稳定结合起来，把优化政府服务与激发市场活力结合起来，使我市经济呈现“一季度罕见下行、二季度降幅收窄、三季度逐步回升、四季度企稳向好”的逐季好转态势。1月份GDP降幅高达20.2％，一季度降幅收窄至5.1％，上半年恢复正增长，三季度增长4.1％，全年增长6.6％，比年初回升26.8个百分点。及时出台了《珠海市企业和企业经营者权益保护办法》等30多个政府规章和文件，着力优化应对金融危机的政策环境。投入资金5亿元支持企业开展科研、技改等活动，发放援企稳岗补贴8060万元，减轻企业负担近5亿元，着力扶持企业发展。财政安排“四位一体”政府风险准备金6000万元，撬动贷款10亿元，着力缓解中小企业融资困难。大力扩内需、促消费，服务业成为经济增长的首要推动力，贡献率达到80.4％；珠海机场年旅客吞吐量、货运量分别为138.6万人次和1.4万吨，两项指标都增长23.5％。积极扶持企业抢抓订单，促进加工贸易转型升级，全年进出口总额实现374.4亿美元，其中出口177.8亿美元，降幅分别由一月份的39.8％、26.8％收窄至20.1％、16％。由于个别大型出口工业企业产值锐减，实际降幅超过30％，全市规模以上工业增加值被拉低7.4个百分点，仅完成478.6亿元，增长1.2％，与上年基本持平。

　　经济结构调整和自主创新成效明显。三次产业增加值比重由上年的2.9：54.6:42.5调整为2.8：51.7：45.5，服务业占GDP的比重提高了3个百分点。初步形成了家电电气、电子信息、精密机械制造、生物医药、电力能源、石油化工、软件和集成电路设计、打印耗材等产业集群。高栏港经济区成为省重化产业集群升级示范区，高新区成为广东省现代信息服务业重点园区，航空产业园成为国家高技术产业基地和省市共建先进制造业基地。以装备制造业为代表的先进制造业发展势头良好，优特电力入选全省50家装备制造业骨干企业，华冠电子等3家企业入选全省100家重点培育装备制造业企业，格力电器等6家企业入选广东省工业企业100强，兴业太阳能等3家企业入选福布斯中国潜力企业200强。预计文化产业产值355亿元，增长22％。产业发展水平不断提高，财政一般预算收入占GDP比重达到10％左右，居于珠三角各市前列。高新技术产业蓬勃发展，预计全年高新技术产品产值950亿元；生物医药和一批民营高科技企业逆势增长，生物医药产业产值达到76亿元，增长12.2％；新认定国家高新技术企业59家。发明专利申请量、授权量达到2366件和1738件，分别增长18％和6.2％。新增2家省部产学研结合示范基地、1家国家级企业技术中心；21个产品被认定为广东省自主创新产品，省级创新型企业增至15家，知识产权优势企业增至36家。健帆生物科技荣获2009年国家科技进步二等奖。实施“以质取胜”、“商标品牌”、“名牌带动”和标准技术战略，我市企业参与制订和修订国际、国家标准151项，国家打印耗材质检中心建设稳步推进，“中富”被认定为“中国驰名商标”。

　　掀起了新一轮发展热潮。高栏港经济区建设日新月异，港口建设和临港产业发展加快推进，地区生产总值、工业增加值、财政一般预算收入、固定资产投资、引进内资注册资本金、港口吞吐量等主要经济指标均实现高速增长。高新区坚持产业发展与新城建设并举，着力打造“创意唐家”品牌，滨海科技新城一期填海造地工程加紧建设，金山软件园动工，巨人网络南方总部暨研发基地奠基，高端产业聚集效应日渐明显。香洲区打造“香洲服务”品牌成绩可喜，服务业占GDP比重提高到56％。金湾区、斗门区以园区整合为契机，促进航空产业园和富山工业园产业发展初见成效。保税区、跨境工业区和万山区发展势头良好。

　　二、加快城乡建设，人居环境日益改善

　　城乡规划和基础设施建设加快推进。发挥规划在城市建设中的先导作用，已完成30多项规划编制工作。交通、能源、防灾减灾、环保和供水工程完成投资11.4亿元，增长25％。维修翻新30座桥梁，新建37座候车亭。拱北口岸客车通道“一站式”电子验放系统工程和横琴口岸扩建工程竣工使用。电网工程完成投资15.6亿元，500千伏国安输变电工程等15个电网项目竣工投运。

　　生态建设不断优化。在全省“环保目标责任考核”中荣获“优秀”称号，“城市环境综合整治定量考核”总分名列全省前茅，连续三年完成减排目标。开展生态城市建设规划研究，制定生态建设四个百分百行动方案，修订《珠海市环境保护条例》。加快推进全国生态文明建设试点工作，通过国家园林城市复查。积极推进36项国家环保模范城市“迎检重点工程”，拱北污水厂改扩建工程完工，南水、三灶水质净化厂建成启用。前山河综合治理工程顺利推进，相关配套工程如期完成。加快城区排洪渠截污分流改造，完成凤凰河综合整治。整合和盘活存量土地，清理处置闲置土地259宗、总面积18.1平方公里。超额完成年度节能任务，我市被省推荐为可再生能源建筑应用项目示范城市。实施“双十百绿”工程，新改建绿地53.3万平方米，种植树木约70万株，建成开放将军山公园，提升海天公园绿化美化水平。完成情侣路沿线海域清理工作，开展全民清洁和市容整治活动，清拆违规户外广告牌2452宗，改造柠溪、朝阳、三灶唐人街等3个农贸市场，市容市貌明显改观。

　　新农村建设成绩喜人。市财政投入“三农”资金23.9亿元，增长19.5％。出台《珠海市饮用水源保护区扶持激励办法》，对保护区实行财政补助。累计改造华侨农场危房3760户，改造农村公路12条、危桥14座。开发补充耕地3760.3亩，超额完成省下达的任务。农业产业化水平不断提高，新增两家省级农业龙头企业，国家和省级农业龙头企业达到9家。全市农业总产值52.8亿元，增长4.6％。农民专业合作社43家，辐射带动农户5700多户。“白蕉海鲈”成为我市首个国家地理标志产品。完成119条行政村新农村建设规划初步成果。加强海洋综合开发和保护，在全省率先完成海岸保护与利用总体规划。南海生物放流活动和首届海洋博览会在我市成功举办。

　　三、加快社会事业发展，改善民生成效显著

　　加大民生投入。全年完成政府投资72.3亿元，增长29.8％，完成投资计划的103.7％。市财政一般预算对教育、社保就业、医疗卫生、文化事业、环境保护、农林水事务等六项民生总投入50.5亿元，增长20％；民生支出占一般预算支出的41.6％，比上年提高1.8个百分点。统筹区域发展，加大对西部地区投入，全年对西部地区教育和医疗卫生、社保就业、环境保护、公共交通、防灾减灾、农林水利、供水设施改造等方面共投入21.8亿元。

　　就业和社会保障水平不断提高。城镇新增就业岗位40877个，城镇登记失业率为2.8％。推进“百企扶百村就业工程”，本市农村劳动力转移就业6779人，吸纳粤东西北地区农村劳动力6506人。为大学生见习、就业和创业提供服务，本市生源高校毕业生就业率达91％。实施适龄青年百分百接受技工教育、百分百实现技能就业工程，5882名普通初高中毕业生免费入读市高级技工学校和中等职业学校，中职毕业生一次性就业率超过98％。社会保险参保人数达389.5万人次，新增17.7万人次。建立社会基本医疗保险普通门诊统筹制度，提高医疗保险待遇水平。出台《珠海市最低生活保障实施办法》，提高重点优抚对象补助标准。农民和被征地农民全部参加医保，12.4万人参加养老保险，参保覆盖率为85％，养老保险参保补贴比例由20％提高35％。海岛居民乘船享受60岁以上老人免费、中小学生6折优惠。

　　文教卫体等社会事业实现新发展。教育发展取得新成绩，14.7万人享受免费义务教育和高中阶段免学费教育，普通高考总上线率为86％，高校全日制在校大学生超过10 万人。实施中小学校舍安全工程，加固校舍65栋。积极解决中小学代课教师问题，已招录代课教师216人。学前教育加快发展，新增3所省一级幼儿园。成功举办珠澳青少年大联欢、第三届民间艺术大巡游等系列活动。重大文化工程项目加紧推进。出台关于镇卫生院改革与发展的意见，镇卫生院改造顺利推进，121个农村卫生服务中心竣工。有效防控甲流等重大疫情，最大限度地保障了居民健康。连续21年完成人口计划控制任务。外经贸、经贸、港澳、外事、侨务、对台和口岸查验等工作在扩大开放、促进合作中发挥了重要作用。新闻出版、民族宗教、体育、统计、国安、海防、打私、打假、人防、气象、科普、仲裁、档案、方志、港澳流动渔民等工作取得新进步，老龄、福利、红十字、慈善和残疾人事业健康发展。

　　社会保持和谐稳定。推进社会治安打防管控，全市社会治安持续平稳。建成镇街综治信访维稳中心23个，入选“2009年中国最安全城市”。圆满完成迎国庆、庆回归十周年等重大活动的安保工作。安全生产形势保持稳定，事故宗数、伤亡人数和直接经济损失同比分别下降20.8％、20.1％和24.1％，连续三年火灾事故零死亡。加强应急管理体系建设，应急管理水平得到进一步提升。食品药品、产品质量安全、打击假冒伪劣和传销违法活动等工作扎实推进，工商、质监、食品药品监督等部门有效维护了市场经济秩序。香洲区成为“全国和谐社区建设示范城区”、“全国平安建设先进区”，拱北街道成为“全国和谐社区建设示范街道”，三灶镇三灶社区成为“全国和谐社区建设示范社区”，吉大海大社区成为“全国综合减灾示范社区”。

　　对口援建帮扶和“双转移”工作扎实推进。我市已完成对口援建工程任务的80％，绵虒小学、汶川县福利院、羌峰（珠海）侨心村等一批援建工程建成启用。市财政预算安排对汶川援建和帮扶资金1亿元。全年对重庆巫山、巫溪、奉节三县和揭阳市揭西县投入帮扶资金1700多万元，并与普宁市、揭西县、惠来县85个村建立了“规划到户、责任到人”的帮扶关系，全面开展对口扶贫开发工作。揭阳、茂名产业转移园被评为省示范性产业转移工业园，分别竞得省财政专项资金5亿元，市财政也按省的要求安排了专项资金1亿元推进产业转移园基础设施建设，两园已竣工项目23个，意向入园项目100多个。

　　四、推动政府职能转变，体制改革迈出新步伐

　　政府机构和审批制度改革继续深化。完成政府机构改革工作，市政府工作部门精简至27个，精简幅度约为1/3，议事协调机构减少83.3％。简政放权，规范审批权限，明确规定除177项审批事项须上报市政府以外，其余事项由各部门依法办理。出台《珠海市建设工程项目行政审批管理办法》，优化审批流程，缩短审批时间，提高基建项目审批效率。

　　社会管理体制改革稳步推进。根据《规划纲要》和省委省政府的明确要求，开展社会管理综合改革试点。率先启动政府购买服务、慈善救助、流动人口服务管理等改革试点，设立促进就业、医疗卫生、小区管理三个咨询委员会，在翠香街道和三灶镇等地推进社区民主自治试点，赋予部分行业协会和社会组织相应的管理职能。

　　金融和国企改革取得新成绩。全市上市公司总数达到20家。珠光集团完成债务重组，削债总额50亿港元。珠海商业银行引入央企战略投资25亿元成功重组。农村信用社改革取得阶段性成果，15亿元中央银行专项票据顺利兑付。推进国有企业改革，通过资源整合等方式组建交通、水务、城建、公交等14家市属国有企业集团。发挥市管国有企业投融资平台作用，得到金融机构大力支持，成功融资498.5亿元，为重大项目建设提供了资金保障。

　　法治、效能和廉政建设得到加强。自觉接受市人大和市政协监督，认真听取各民主党派、工商联、各人民团体及无党派人士的意见建议。提请市人大常委会审议《珠海经济特区科技创新条例》等地方性法规草案8件，已颁布《珠海市经济特区政府投资项目管理条例》等3件。制定《珠海市建设工程招投标管理办法》等政府规章6件。提请市人大常委会审议各项议案和工作报告62项。市政府高度重视“两案一议”办理工作，6件市人大议案办理工作加紧推进，165件代表建议和336件政协提案全部办结，被省政协评为2009年度“承办提案先进单位”。强化审计监督、效能监察、廉政监察、执法监察和纠风工作，开展政风行风民主评议和创建群众满意基层站所活动，加大案件查处力度，反腐倡廉工作得到加强。完善公众咨询、窗口办事、部门责任白皮书等制度，加强监督考核和行政问责。切实做好政府重大决策及其落实情况等重点信息公开，政务公开工作在全省考核中获得良好成绩，我市荣获中国政府网站“优秀奖”和“信息公开领先奖”。政务环境不断优化，入选港澳台和大陆最具投资潜力十大城市，在广东省社科院全省地方政府公共服务公众评价调查中总体满意度名列第一。

　　回顾一年来所取得的成绩，让我们倍感自豪；党中央、国务院和省委、省政府对珠海的高度重视和支持，使我们倍受鼓舞；全市各级各部门和广大干部群众迎难而上、拼搏进取，让我们倍增动力。尤其是去年底胡锦涛总书记视察珠海，明确要求我们深入贯彻落实科学发展观，坚定不移调结构、脚踏实地促转变，全力打好转变经济发展方式这场硬仗，为珠海又好又快发展指明了前进的方向；温家宝总理主持召开国务院常务会议，审议通过《横琴总体发展规划》，盼望已久的横琴岛开发已经启动，为珠海发展再添新亮点；李克强副总理出席港珠澳大桥动工仪式并视察珠海，汪洋、黄华华等领导多次到珠海指导工作，带来了党中央国务院和省委省政府对珠海的亲切关怀和巨大支持，进一步增强了珠海人民锐意进取、开拓创新的信心和决心。在此，我代表市人民政府，向各级领导和部门，向各位人大代表、政协委员，向各民主党派、工商联、各人民团体及无党派人士，向奋斗在全市各条战线的广大干部群众，向中央和省驻珠单位、驻珠军警部队官兵，向各位老领导、老同志，向所有关心和支持珠海发展的港澳台同胞、海外侨胞、国际友人和社会各界人士，表示崇高的敬意和衷心的感谢！

　　在看到成绩的同时，我们也清醒认识到发展中迫切需要解决的问题：我市经济总量偏小，交通等瓶颈制约仍未得到根本解决，核心城市辐射和带动功能有待增强；重大项目推进还不快，投资规模不大；自主知识产权、自主品牌的企业和产品不多，自主创新能力有待提高；市容市貌与广大市民要求仍有差距，城市规划建设管理面临新课题；政府执行力仍有待提高，行政效能建设任重道远。对这些问题，我们必须高度重视，有针对性地采取有效措施，切实加以解决。

　　前景美好、乘势而上的2010年

　　2010年，是珠海经济特区成立30周年，也是实施“十一五”规划的最后一年。做好全年的工作，意义十分重大。综观国内外形势，虽然全球金融危机的影响仍未根本消除，经济复苏将是一个缓慢曲折的过程，外部发展仍有许多不确定因素；我市经济回升的基础仍不牢固，保持经济平稳较快发展面临较大困难，转变经济发展方式任务艰巨。但是，我们也面临着难得的发展机遇、具备了良好的发展基础、迎来了强劲的发展势头，前景光明而美好。我们的重大机遇在于党中央国务院和省委省政府对珠海的高度重视和亲切关怀，在于《规划纲要》的全面实施和区域合作的深入推进，在于港珠澳大桥等重大交通项目的带动效应。我们的良好基础在于得天独厚的资源禀赋和日趋完善的基础设施，在于历届市委市政府带领全市人民取得的辉煌成就，在于初具规模的产业条件和宜居宜业的城市环境。我们的强劲势头在于一批新的重大产业项目的相继落户和动工投产，在于重点区域开发建设的日新月异，在于城市发展新格局的初步形成。我们一定要抓住机遇、发挥优势、乘势而上，努力推动珠海新一轮大发展。

　　今年市政府工作的指导思想是：深入贯彻科学发展观，全面落实中央经济工作会议、胡锦涛总书记视察珠海重要讲话以及省委十届六次全会和市委六届七次全会精神，以贯彻实施《珠江三角洲地区改革发展规划纲要》和《横琴总体发展规划》为主线，以转变经济发展方式为核心，坚定不移调结构、脚踏实地促转变、扩大内需稳增长、自主创新强产业、改革开放添动力、统筹城乡惠民生，努力在重大项目、产业发展、宜居城市、改善民生、服务型政府建设等方面实现新突破，推动我市经济社会又好又快发展，努力建设珠江口西岸核心城市，争当实践科学发展观的排头兵。

　　今年我市经济社会发展的主要预期目标为:实现地区生产总值增长9％，人均生产总值增长7.9％；规模以上工业增加值增长11％；固定资产投资增长15％；社会消费品零售总额增长15％；外贸进出口总额增长5％；实际吸收外商直接投资额力争有所增长，引进内资注册资本金增长15％；财政一般预算收入增长8％；万元生产总值能耗完成“十一五”规划目标，控制在0.56吨标准煤以内；二氧化硫和化学需氧量排放量分别控制在3.3万吨和3万吨以内；服务业增加值占GDP比重达46.5％；全社会研发（R＆D）投入占GDP比重达1.7％；城镇居民人均可支配收入和农渔民人均纯收入分别增长10％和8％；城镇登记失业率控制在3.5％以内；户籍人口自然增长率控制在8.8‰以内。

　　今年的政府工作要突出抓好六个方面：一是在交通等重大项目建设方面下功夫。着力抓投入、上规模，开展“建设大交通、狠抓大项目”活动，全力推进一批重大交通和产业项目建设，增强核心城市聚集带动能力，加快珠江口西岸交通枢纽城市建设。二是在“双提升”方面下功夫。加快现代产业体系建设，坚持先进制造业和现代服务业“双轮驱动”，积极培育战略性新兴产业，提升产业竞争力，提升自主创新能力。三是在统筹城乡发展方面下功夫。以科学的规划指导城乡建设和发展，推进生态示范区建设，加强生态环境保护。四是在深化改革开放方面下功夫。进一步巩固机构改革成果，加大社会管理体制改革步伐，以改革开放促发展。五是在保障和改善民生方面下功夫。坚持以人为本，把解决人民群众最关心、最直接、最现实的利益问题摆在更加突出位置，加快发展社会事业，推进基本公共服务均等化，让老百姓充分享受改革发展成果。六是在提高政府执行力上下功夫。加强学习，提高抓落实的能力和本领。明确职责、落实责任，构建有利于执行的体制机制，推动各项工作落实。

　　为此，今年要重点做好以下工作：

　　一、积极实施《规划纲要》，增强区域发展竞争力

　　加快横琴新区建设步伐。横琴新区开发建设是中央交给我们的一项重大任务，也是我市未来发展的新亮点。要落实横琴市级管理权限，加紧编制横琴新区基础设施、产业发展、城市建设、环境保护、横琴口岸分线管理等专项规划。加大横琴市政道路、桥梁、口岸出入境客货车通道“一站式”电子验放系统等基础设施投入和建设力度。加强协调沟通，争取上级支持，尽快落实相关配套政策。加大项目推进和土地整合力度，推动长隆国际海洋度假区、多联供燃气能源站等项目加快建设，大力发展商务服务、休闲旅游、科教研发和高新技术产业，努力推动“一国两制”下探索粤港澳合作新模式的示范区、深化改革开放和科技创新的先行区建设，逐步使横琴新区成为促进珠江口西岸地区产业升级的新平台。

　　加快西部地区开发步伐。西部地区是我市未来发展的希望所在，要以大投入带动大开发、以大项目推动大发展。今年市政府对西区投入基建资金22亿元，用于重大交通、环境保护、防灾减灾和农村设施等22个项目，占市政府年度基建投资总额的48.3％，剔除不可比因素，比上年增加6.7亿元。围绕建设“世界级船舶和海洋工程装备制造基地、国家级石油化工和清洁能源基地、区域性港口物流中心”的目标，推动高栏港经济区跨越发展。尽快完成中国海油陆上终端项目平基工程建设，加快建设中船相关配套工程，全面推进装备制造区和石化区配套基础设施建设。推进市政基础设施建设，积极推进西湖城区规划建设，加快平沙新城填土工程和斗门城区“一河两岸”建设，规划建设西部中心城区。以加快城镇化进程推动新农村建设，做大做强村集体经济，引导和扶持34个欠发达村发展，确保欠发达村集体经济年收入增长30％以上。

　　加快东部地区发展步伐。东部地区要继续在转型提升上下功夫，加快我市现代服务业发展。要按照“扩大总量、提升质量、发挥优势、突出重点”的要求，优先发展金融保险、现代物流、信息服务、会展、科技研发、工业设计、总部经济等生产性服务业，壮大发展旅游休闲、商贸餐饮、社区服务、家政养老服务等生活性服务业，培育发展数字出版、文化传媒、动漫创意等新兴潜力服务业，形成特色鲜明、布局合理、优势互补的现代服务业发展格局。稳妥推进服务业对外开放，承接国际软件和商务服务外包。加速推进国家打印耗材质检中心、联想纳思达打印机研发制造基地、格力大金模具研发中心、高新区互联网产业园等项目规划建设。积极引进一批总部基地项目，争取一批国家级和省级现代服务业重点项目落户香洲，加快香洲转型步伐。有序推进裕华聚酯等企业向西部园区转移。加快建设保税物流平台，促进展览、展销功能集聚，推动跨境工业区向珠澳跨境经济合作区转型。加大海洋综合开发力度，加快推进海岛客运站场、桂山油库二期扩建、东澳岛旅游等项目建设，力争在海岛旅游业、海洋渔业、仓储物流、海洋科技产业发展和新能源项目引进方面实现新突破。

　　加快区域一体化步伐。推进区域一体化是落实《规划纲要》的基本要求，我们要努力完成省下达的年度目标任务。加快与珠三角其它城市基础设施、产业布局、城乡规划、公共服务和环境保护一体化步伐，积极推进珠中江通信同城化等各领域合作。加大CEPA实施力度，落实服务业扩大开放政策在珠海先行先试，扩大珠港澳在文教卫、环保、社会服务等领域的合作，积极推进珠澳同城化。年内建成竹银水源工程，启动第二条珠澳供水管道规划建设，保障珠澳供水安全。加强珠澳口岸合作，积极探索口岸便捷通关模式，加快推进拱北口岸扩建工程。

　　二、大力抓好重大项目，增强内需对经济增长的拉动力

　　着力突破项目瓶颈制约。项目瓶颈制约是去年项目建设的主要问题之一。要主动查找制约项目建设存在的问题，突出针对性，推动重大项目建设。加快项目前期工作，力争项目早申报、早立项，保证重大工程项目如期动工建设。资金是项目建设的保障，要通过市财政预算安排、多渠道筹资、融资以及争取上级支持等方式，千方百计落实重大项目建设资金。严格落实土地利用总体规划，建立用地报批协同审批长效机制，着力解决项目用地制约。编制完善我市海洋功能区划和区域性用海规划，着力破解项目用海难题。健全项目协调机制，建立项目专家咨询制度，加强项目建设相关政策法规研究，优化基建项目审批程序，提高项目决策的规范性、科学性、前瞻性。进一步明确审批部门、项目主管单位与建设单位之间的职责，落实重大项目责任制，加强项目督查督办，形成加快推进项目的强大合力。

　　着力抓好项目引进。投资是推动发展的重要动力。要坚持招商选资，采取产业招商和专业队伍上门招商等多种方式，进一步创新招商手段，拓宽招商领域，优化招商环境，加快引进一批优质项目。继续瞄准世界500强、大型央企和民营科技企业，实现引进内资与外资并举，引进项目与增资扩产并举。开展对重点区域、重点行业、重点企业和战略性新兴产业的招商活动。强力推进中国海油深水工程基地、南海天然气陆上终端、LNG接收站、天然气发电、玉柴机器等重大项目建设，力争宝塔石化首期、成城沥青、中石油物流中心、汉胜科技二期等一批项目尽快投产，力争港口工程装备、丽珠制药二期、银通新能源、方正科技二期、数控机床等一批项目动工建设。总投资3130亿元的“十大重点建设工程”年内计划投资260.2亿元，增长88.7％。市政府投资项目102项，计划投资规模为80.2亿元，增长15％。其中，新开工项目45项，续建项目57项。优化投资结构，积极激活民间投资，引导资金投向基础设施、自主创新、环境保护和改善民生等重点领域，全力推动一批项目动工和投产。

　　有效扩大消费需求。扩大内需是推动经济增长的内生动力。抢抓重大项目全面开工建设的大好机遇，鼓励重大项目建设在珠海采购。积极开展珠海名优特产品外省行活动，深度开拓国内市场。多渠道增加居民收入，增强居民消费能力。全面推进汽车、家电、农机“三下乡”和汽车、家电以旧换新活动，完善农村流通网络、扩大城乡消费规模。加强商业网点规划建设，加强消费信贷服务，扩大文化娱乐、信息服务、住房家政、教育培训、旅游休闲和健康产业等领域的消费，增强消费对经济增长的拉动作用。保持房价基本稳定，支持居民自住和改善性购房需求，推动房地产业健康有序发展。

　　三、强力推进交通先行，打造交通枢纽城市

　　加大交通建设力度。举全市之力，加快推进快速路网、片区路网等交通基础设施建设，逐步实现市内任何一个地方都可以在30分钟内上高速。完成已开工的珠海大道一期、柠溪路、紫荆路、翠香路等道路改造和板樟山隧道大修工程，打通凤凰山公路隧道。推进港珠澳大桥珠澳口岸人工岛、广珠铁路、广珠城际轨道、高栏港高速、机场高速、井岸二桥等一批项目加快建设。动工改造珠海大道二期、港湾大道、港昌路、明珠路等市政道路。力争年内动工建设连接东西部的金海大桥，完成金港、香海高速公路申报立项，开展机场北路北段工程前期工作。推进港珠澳大桥珠海连接线、广珠城际轨道延长线、广珠西线珠海段等的规划建设。

　　提升双港集疏运能力。双港是我市发展的强劲引擎。大力推进高栏港区10万吨级进港主航道疏浚、铁炉湾防波堤等项目，加快10万吨级煤码头、15万吨级矿石码头、10万吨级集装箱码头和南水作业区弃土岸壁整治工程建设，完善港口基础设施。积极引进大型航运企业，加快开通国际集装箱班轮航线。加紧万山港区功能定位研究和规划编制。完善珠海机场配套路网，引入大中型民用客机运营，增强珠海机场吸引力和区域竞争力。大力发展航空货运，培育和壮大空港物流产业。鼓励航空公司在珠海设立基地，有效发挥空港作用。

　　加强城市交通管理。完善交通规划体系，抓紧编制城市综合交通运输体系规划和城区轨道交通规划，修订完善现有交通专项规划，科学规划建设好与港珠澳大桥相匹配的交通路网。以缓解交通拥堵、预防重特大交通事故为目标，着力改善交通状况。加大出租车、中小学校巴、危险品运输及其它运营车辆的监管，开展交通秩序综合整治，净化路面交通环境。完善交通信号智能控制系统，科学规划城市主干道交叉口，加快规划建设人行过道，提高路网总体通行能力。实施公交优先战略，建设绿色公交。提高城市交通科学管理能力，构建科学、畅通、有序、安全的城市交通格局。

　　四、加快建设现代产业体系，增强产业竞争力和自主创新能力

　　抓园区强载体优存量。园区是构建现代产业体系的主战场。加大工业园区基础设施配套力度，进一步完善项目发展环境。制定分类指导的园区产业政策，推动产业集群化、特色化、生态化发展，促进产业一体化布局。加快推进中航通用飞机产业基地建设，加强以通用飞机制造、总装、维修、运营等为主的通用航空产业发展。加大航空产业招商力度，千方百计引进相关项目落户航空产业园。大力引进和培育游艇及其配件制造业，发展游艇服务业，增强平沙游艇产业基地聚集能力。围绕培育战略性新兴产业，加快富山工业园装备制造、临港加工产业发展。落实加快珠海高新技术产业开发区发展的意见，高新区要“增速进位”，全力引进一批、建设一批、升级一批高新技术产业项目，新增一批国家级高新技术企业，努力实现高新技术产业发展的新突破。

　　聚人才促创新强后劲。推动自主创新，企业为主体，人才是关键。完善人才引进政策，大力吸引海内外高层次人才，引进科研创新团队。加强人才教育培训体系建设，不断提高专业人员的创新能力。落实人才落户、就医、子女入学、住房等方面的优惠政策，搭建高层次人才创业平台，优化人才发展环境。依托高新区科技企业和高校，积极创建国家级创新型科技园区。支持优势企业申报工程技术中心、企业技术中心、博士后流动站，支持企业开展技术改造和创新。鼓励中小企业参与省部企业科技特派员行动计划，促进产学研合作。加强知识产权保护，继续实施“技术标准”、“名牌带动”和“以质取胜”战略，培育名牌名标产品，推动“珠海制造”向“珠海创造”转变。

　　调结构促转型助升级。坚持“好”字当头，壮大优势产业，大力发展高端制造业、高端服务业、高新技术产业，改造提升传统产业，培育发展战略性新兴产业，推动产业结构调整。实施“7·50工程”，重点培育50个战略性新兴产业项目，50个先进制造业项目、50个现代服务业项目、50个优势传统产业项目、50个现代农业项目、50个自主创新项目、50个名牌名标项目，引领我市现代产业体系建设。大力推进新材料、生物医药、研发服务、海洋资源开发和综合利用等新兴产业发展。加快海洋工程装备制造、造船、游艇等产业及其配套产业基地建设。办好第八届中国航展，努力在办展模式、航展规模、装备展览等方面取得新突破。强化国有资产运营监管，发挥国有资本在基础设施、公用事业和关键行业的主导作用。完善中小企业服务体系，加大民营企业扶持力度，发展壮大民营经济。促进加工贸易转型升级，扶持一批有规模、有优势的加工贸易企业向自主设计、自主品牌转型。完善扩大内销政策措施，着力扩大企业内销规模。抓住中国-东盟自由贸易区正式建立等机遇，积极打造珠海产品展示平台，拓展东盟等国内外新兴市场。加大产业和劳动力“双转移”工作力度，努力完成省交给我市的“双转移”任务。

　　五、统筹城乡区域发展，增强宜居城乡魅力

　　强化规划龙头导向作用。做好“十二五”规划编制工作，深入开展重大课题研究，加紧编制专项规划、区域规划等各类规划。按照主体功能区规划的要求，明确优化开发、重点开发、限制开发和禁止开发区域，引导经济布局、人口分布与资源环境承载能力相适应。加快推进第五轮城市总体规划修编，制定宜居城乡建设行动纲要。科学统筹各类规划，确保经济社会发展规划、土地利用总体规划和城市建设规划“三位一体”、有机衔接。严格执行《珠海市城市规划条例》，坚持“先规划后建设”，依法查处违法建设行为，维护城乡规划的严肃性和权威性。提高我市城乡规划透明度，鼓励市民参与城乡规划。

　　着力保环境、优生态。生态环境是我市重要的城市品牌，要继续落实四个百分百行动方案，积极开展生态文明示范市创建活动。加大节能减排力度，确保完成“十一五”节能减排目标任务。做好能源发展规划，加大重点能耗企业能源计量监管力度。开展低碳经济试点，鼓励清洁生产、资源综合利用，发展绿色环保产业和循环经济。做好迎接环保模范城复查工作，确保完成各项迎检工程。建设茶冷迳固体废物处理等项目，完成医疗垃圾集中处置设施的建设，完成黄杨山、平沙简易垃圾填埋场的封场工作，加快推进万山海岛垃圾处理项目建设。借鉴先进地区经验，积极推进垃圾分类和资源化处理。建立健全环境污染突发事件应急体系，强化危险废物污染防治、污染事故控制和监测预警工作。完善城市污水管网，加大西部地区污水处理设施建设力度，完成新青、富山、白蕉、平沙水质净化厂工程建设。加快推进前山河综合整治工程，完成前山北部排污排洪主体管网工程。大力推进“双十百绿”工程，加快山体整治复绿工作，开展全民清洁行动，提升城市绿化、净化、亮化和美化水平。按照“一年基本建成，两年全部到位，三年成熟完善”的要求，遵循“统一规划、以人为本、因地制宜、市区联动”的原则，大力推进我市绿道网规划建设，打造路景结合、特色鲜明、功能明显的区域绿道网，提高城市生态建设水平。

　　着力推进“三旧”改造。认真贯彻落实省政府《关于推进“三旧”改造促进节约集约用地的若干意见》，按照“政府引导、市场运作、节约集约、统筹规划、明晰产权、尊重历史”的原则，采取改建、拆建、收购和收回等方式，逐步铺开旧城镇、旧厂房、旧村庄改造工作。出台政策措施，抓紧编制规划，明确改造规模、地块和时序，注重严格管理和规范实施，确保改造工作依法依规、平稳有序推进。在“三旧”改造中，首先要落实“退二进三补公”措施，促进“三旧”改造与宜居城乡建设的有机结合。

　　加快新农村建设步伐。新农村的建设是我市现代化建设的重点，也是难点。要加大财政投入，整合涉农资金，集中财力，加快推进新农村建设。市财政投入8亿元用于海堤达标加固、乾务水库排洪渠整治、天生河水闸和西安泵站重建、省道S365线中心涌至井岸二桥段等工程建设。大力推进农村水改、厕改和污水处理达标工程。落实强农惠农政策，今年市财政投入农林水事务3.6亿元，增长27.4％。调整农村经济结构，积极发展现代农业，加快建设生态农业园区，办好台湾农民创业园。推进农业标准化、产业化、集约化发展，实行农产品标识管理和市场准入制度，发展无公害农产品、绿色食品和有机食品。加强农业科技推广和培训，逐步完善农业信息服务体系。扶持农业龙头企业，优化农产品流通环境，多渠道增加农民收入。着力深化农村综合改革，深入推进集体林权制度改革。加强农村社区建设，丰富农村文化生活。深化平沙、红旗、白藤华侨农场改革，加快危房改造步伐，进一步改善困难群众生产生活条件。认真落实推动海洋经济科学发展、建设海洋经济强市的实施意见，实施科技兴海战略，推进海洋综合开发。

　　做好对口援建帮扶和“规划到户、责任到人”扶贫开发工作。今年市财政安排汶川援建资金1.1亿元，切实落实重庆三县和揭阳市对口扶贫资金。采取产业扶贫、智力扶贫、劳务扶贫、旅游扶贫等综合扶贫措施，继续做好汶川绵虒镇和重庆三县对口帮扶工作，派驻三个工作组进驻揭阳协调推进扶贫开发工作，全面落实“规划到户、责任到人”扶贫开发措施。

　　六、继续深化体制改革，增强经济发展动力和活力

　　巩固政府机构改革成果。按照“权责一致、重心下移、减少层次”的原则，大力简政放权，扩大区政府（管委会）和镇政府管理权限。巩固大部门体制改革成果，缩短磨合期，切实转变职能，形成科学、高效、协调的政府运行机制。深化行政审批制度改革，清理审批事项，力争非行政许可审批事项减半。简化审批程序，积极推行网上审批。加大行政服务中心建设力度，实现行政审批事项百分百进驻窗口。全面推进事业单位岗位设置和人员聘用工作，深化人事制度改革。

　　加快社会管理体制改革步伐。通过财政扶持、特许经营、政府购买服务等形式，培育发展社会组织。推进社会工作人才队伍建设试点工作，探索“社工＋义工”联动服务模式。加快社区社会组织建设，完善市、区、镇（街）三级社会管理服务体系。加大对城市社区服务设施的投入力度，不断改善服务条件。健全农村社区基本公共服务体系，建立政府行政管理与基层群众自治互动衔接机制。全面推行“一证通”制度，提高对流动人口的服务水平。

　　深化城管和土地管理体制改革。按照责权利相结合的原则，探索更加有效的城市管理体制。实行网格化、精细化、规范化管理，坚决查处各种违法违章行为，提升城市管理水平。实施《珠海市建立土地执法共同责任制度的规定》，依法使用土地，推动节约集约用地。积极盘活土地资源，加强耕地保护。继续做好利用园地、山坡地开发补充耕地工作，继续开展围海造地先行试点。完善农村征地补偿制度，健全被征地农民的社会保障和就业安置机制。

　　稳妥推进其它各项改革。加强国有企业监管，完善国有企业投资管理制度。加强金融监管，积极防范和化解金融风险，优化金融生态环境。继续推进跨境贸易人民币结算试点工作，大力建设金融创新服务区。搞好村镇银行、小额贷款公司等新型农村金融机构试点。创新中小企业融资模式，推进产权交易中心建设，实现信贷市场与产权市场互动发展。注重培植财源，完善社会综合治税体系，营造公平的税收环境。健全公共财政体系，优化财政支出结构，推进财政预算和资金绩效管理改革，完善激励型财政机制。按照事权、财权相匹配的原则，深化财政管理体制改革。

　　七、着力惠民生、强保障，努力构建和谐社会

　　加大就业和社会保障力度。今年社保和就业投入11.5亿元，增长13.1％。实施积极的就业政策，鼓励有实力的大企业创造更多的就业机会。加强对困难人员的就业援助，大力促进高校毕业生就业。实施创业带动就业工程，鼓励和支持劳动者自主创业。继续实施“双百工程”，深入开展创建“充分就业村”活动，加强人力资源社会保障服务平台建设。加大劳动保障监察执法力度，切实维护劳动者的合法权益。健全社会保障制度，进一步扩大社会保险覆盖面。优化整合社会基本医疗保险制度，扩大城乡医疗救助范围，增加救助病种。落实最低生活保障制度，实行分层分类施保，切实做到应保尽保。完善农民和被征地农民养老保险制度。加快廉租住房等保障性住房建设。

　　加快发展文化教育卫生体育事业。今年市财政对文化传媒、教育、医疗卫生投入为1.8亿元、24.2亿元和5.3亿元，分别增长15.1％、8.3％和9.9％。全面开展创建全国文明城市活动，开展国家历史文化名城申报工作。大力开展文体旅游活动，壮大文化、旅游和体育产业规模。开展全民健身活动，加大体育设施建设力度。加强公共文化设施和重大文化项目建设，加强历史人文资源保护与开发，进一步增强市民认同感和归属感，提升城市文化软实力。研究制定中长期教育发展规划，推进各类教育协调发展。继续实施中小学校舍安全工程，加快建设职业教育基地，启动金湾一中规划建设，继续推进市一中、市实验中学、斗门一中、斗门和风中学等扩建工程。继续办好大学园区，推进高校与企业紧密合作。深化医药卫生体制改革，完善覆盖城乡的基本医疗卫生制度，稳妥推进公立医院改革试点。加强重大疾病防控工作，提高医疗服务和管理水平。继续推进市人民医院北区、市第二人民医院二期、市疾控中心和市传染病专区建设，开展市第二中医院规划建设的前期研究，完成镇卫生院改造任务。

　　切实维护社会安全稳定。强化食品药品安全、产品质量和安全生产监管，遏制重特大公共安全事故发生。妥善处理城市发展中遇到的各种问题，重视人民调解工作，加强法制宣传教育，引导群众依法、理性表达诉求，及时化解社会矛盾。加强法律援助，加快建设公益性法律服务体系。集中力量解决影响社会和谐稳定的源头性、根本性、基础性问题，严厉打击各种违法犯罪活动。健全信访维稳工作责任制，加强镇街综治信访维稳中心建设，加大社会矛盾纠纷排查调处力度。加强应急管理体系和保障能力建设，完善海上救助协调等应急机制。

　　推动各项社会事业全面发展。加强人口计生服务和管理，稳定低生育水平，做好第六次人口普查工作。继续做好民族宗教、档案、人防、仲裁、打私、海防、气象、地震、地质、方志、港澳流动渔民和科普工作。加快发展妇女儿童、红十字、慈善和残疾人事业。增强市民国家安全意识和国防意识，加强双拥共建和民兵预备役工作。支持中央和省驻珠单位依法履行职责。

　　八、加强政府自身建设，增强政府执行力

　　推进学习型政府建设。珠海新一轮发展对政府建设提出了新的更高的要求。倡导终身学习理念，深化对科学发展的认识，提高科学发展的坚定性和自觉性。加强职业能力培训，注重学习哲学、历史、法律、科技、管理和信息网络等各方面知识，尤其要结合推进珠海大发展的需要，加强对深水海洋工程装备、通用航空、生物医药、新材料、新能源以及软件等新兴产业知识的学习，不断优化干部队伍知识结构，提高抢抓新机遇、干成大事业的能力和本领，提高推动科学发展的能力和本领，提高推动经济发展方式转变、实现又好又快发展的能力和本领。营造良好的学习风气，大兴调查研究之风，以学习成果指导工作实践，推动珠海新一轮大发展。

　　提高依法行政能力。严格按照法定权限和程序行使权力、履行职责，推进法治政府建设。完善行政执法程序，规范行政执法行为，提高依法行政水平。加强行政复议和行政应诉工作。认真做好人大议案、代表建议和政协提案的办理工作，充分尊重各民主党派、工商联、各人民团体及无党派人士的意见和建议，进一步提高科学、民主、依法决策水平。

　　加强行政效能建设。以“政治坚定、业务精通、清正廉洁、作风优良”为目标，加强公务员队伍建设。加强行政问责和行政监察，健全执法过错、违纪违法责任追究等制度，做到有责必问、有错必究。建立健全政府绩效评估制度，加强电子监察对重点项目审批和行政执法的全方位监督，提高办事效率。坚持说实话、出实招、办实事，在狠抓落实上下功夫，以只争朝夕的紧迫感和责任感，推动珠海升温发展。

　　扎实推进廉政建设。认真落实党风廉政建设责任制，注重加强教育、注重制度建设、注重强化监督、注重案件查处，加大专项治理和纠风工作力度，着力从源头上预防腐败。加大工程建设领域反腐败力度，进一步规范政府采购和工程建设行为。坚持艰苦奋斗，厉行节约。健全政府信息公开制度，构筑信息公开平台，切实保障人民群众的知情权、参与权、表达权和监督权，建设“阳光政府”。

　　各位代表，今年是珠海经济特区成立30周年。30年来，历届市委、市政府带领珠海人民走过了不平凡的历程。认真回顾和总结30周年经济特区建设的辉煌成就和宝贵经验，对于我们抢抓新机遇、迎接新挑战、实现新跨越，具有十分重要的意义。我们要隆重纪念经济特区成立30周年，精心策划多种形式的纪念活动，大力弘扬特区精神，激励全市人民开拓进取、奋发有为，增创特区新优势，履行特区新使命，争当推动科学发展、促进社会和谐的排头兵！

　　新的一年，各项工作任务十分繁重，责任重大而光荣。让我们紧密团结在以胡锦涛同志为总书记的党中央周围，在省委、省政府和市委的坚强领导下，团结带领全市人民，开拓创新，奋发进取，加快珠江口西岸核心城市建设步伐，以优异的成绩向珠海经济特区成立30周年献礼！
